# Offshore exposure experiments on cuttlefish indicate received sound pressure and particle motion levels associated with acoustic trauma

**DOI:** 10.1038/srep45899

**Published:** 2017-04-05

**Authors:** Marta Solé, Peter Sigray, Marc Lenoir, Mike van der Schaar, Emilia Lalander, Michel André

**Affiliations:** 1 Laboratory of Applied Bioacoustics, Technical University of Catalonia, Barcelona, Spain; 2 FOI, Department of Underwater Research, Stockholm, Sweden; 3 INSERM U.1051, Institute of Neurosciences of Montpellier, Montpellier, France

## Abstract

Recent findings on cephalopods in laboratory conditions showed that exposure to artificial noise had a direct consequence on the statocyst, sensory organs, which are responsible for their equilibrium and movements in the water column. The question remained about the contribution of the consequent near-field particle motion influence from the tank walls, to the triggering of the trauma. Offshore noise controlled exposure experiments (CEE) on common cuttlefish (*Sepia officinalis*), were conducted at three different depths and distances from the source and particle motion and sound pressure measurements were performed at each location. Scanning electron microscopy (SEM) revealed injuries in statocysts, which severity was quantified and found to be proportional to the distance to the transducer. These findings are the first evidence of cephalopods sensitivity to anthropogenic noise sources in their natural habitat. From the measured received power spectrum of the sweep, it was possible to determine that the animals were exposed at levels ranging from 139 to 142 dB re 1 μPa^2^ and from 139 to 141 dB re 1 μPa^2^, at 1/3 octave bands centred at 315 Hz and 400 Hz, respectively. These results could therefore be considered a coherent threshold estimation of noise levels that can trigger acoustic trauma in cephalopods.

Levels of introduced anthropogenic underwater sounds have increased significantly over the last century and anthropogenic noise is now recognized as a significant stressor for marine and freshwater fauna. While advances have been made in understanding the effects on marine mammals^1^,^2^,^3^,^4^,^5^, and fishes^6^,^7^,^8^ the impact of noise on marine invertebrates has not received a similar scientific attention. Much remains to be learned about sound sensitivity or sound-producing capabilities of invertebrates, both their response to and the potential effect by man-made sounds. Several authors have addressed studies on invertebrate sensitivity to noise and possible negative effects after sound exposure^9^,^10^,^11^,^12^,^13^,^14^,^15^,^16^,^17^. A detailed literature review on these effects can be found in recent publications^18^,^19^,^20^.

In previous studies on four species of cephalopods, the common Mediterranean cuttlefish (*S. officinalis*), common octopus (*Octopus vulgaris)*, the European squid (*Loligo vulgaris*) and the southern shortfin squid (*Illex coindetii*)^18^,^19^,^20^, we showed that exposure to artificial noise had a direct consequence on the functionality and physiology of the statocysts, sensory organs, which are responsible for invertebrate equilibrium and movements in the water column. The statocyst morphology and its functions have been described elsewhere by different authors^18^,^19^,^20^,^21^,^22^,^23^,^24^,^25^,^26^ as well as its analogy with vertebrate vestibular system^27^,^28^. Figure 1 allows a detailed observation of the statocyst inner sensory systems. The sensory input of the statocyst includes sensitivity to low frequency sound waves up to 400 Hz^24^,^29^,^30^,^31^,^32^,^33^,^34^,^35^,^36^.

As previously described in details in André *et al*.^37^, it was suggested that particle motion could encompass the whole body of cephalopods and cause it to move with the same phase and amplitude: the statolith organs would then be stimulated by whole-body displacements and would act as a harmonic oscillator^29^,^30^,^31^,^32^,^33^,^34^,^35^. When an animal accelerates, the statolith would move, bending the sensory hair cells^37^ thus, the statolith would serve as a receptor of kinetic sound components^29^,^35^. This recent study confirmed that the whole body of scallops and cuttlefish vibrates when stimulated by underwater sound^37^. Novel laser Doppler vibrometer techniques have indeed opened the possibility to measure whole body (distance, velocity and acceleration) vibration, as a direct stimulus eliciting statocyst response, and offered the scientific community a new level of understanding of the marine invertebrate sensitivity to sound^37^. Although these techniques were already applied to other species such as amphibians, reptiles and crustaceans to measure specific organ vibration^38^,^39^,^40^, this constituted the first measurement of the whole body vibration induced by underwater sound that had been performed in any marine organism. Nevertheless, the question remained about the contribution of the experimental setting, and in particular the consequent near-field particle motion influence from the tank walls, to the triggering of the trauma. No quantification of the lesions were performed, nor were threshold levels determined to explain the onset of the trauma. Here, offshore noise controlled exposure experiments (CEE) on common cuttlefish (*Sepia officinalis*), were conducted at three different depths and distance from the source to measure the response of exposed animals to different sound pressure and particle motion levels and quantify the response of the statocyst sensory epithelia.

## Results

### Ultrastructural analysis of the Sepia officinalis statocyst sensory epithelia

#### Macula epithelia

**Figure 2:**
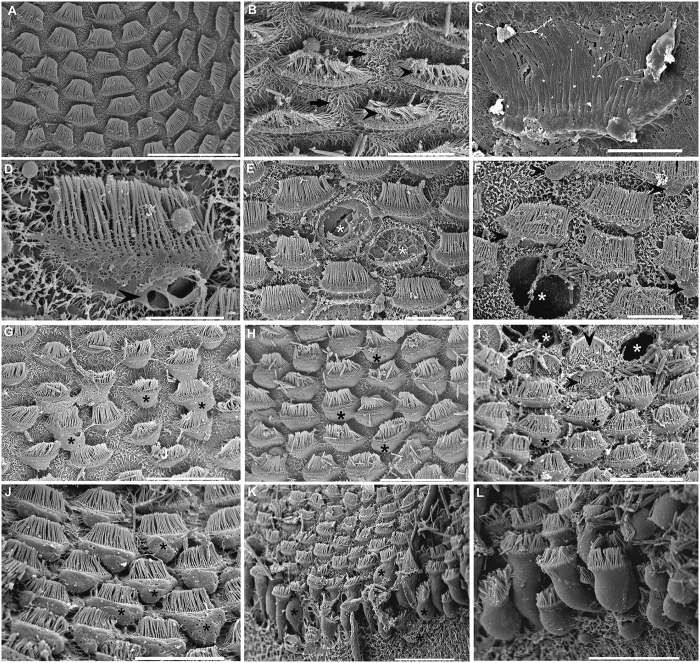
SEM. S. officinalis macula statica princeps (msp). (**A)** control animals. (**B–H)** immediately after sound exposure. (**I**–**L**) 48 h after sound exposure. (**A**) The arrangements of the kinociliary groups of the hair cells in regular lines following the epithelium shape are visible. (**B**) Hair cells present bent and flaccid kinocilia (arrowhead) and disorganized microvilli (arrow). (**C**) A hair cell presents its bundle of kinocilia totally fused. (**D**) A hair cell shows spherical holes on the base and rupture the plasma membrane (arrowhead). (**E**) Some hair cells have lost the bundle of kinocilia (white asterisk). (**F**) A hole on the epithelium due to a hair cell extrusion is visible (white asterisk). (**G**, **H**) The apical poles of the hair cells extruded above the epithelium in the statocyst cavity are visible (black asterisk). In G some kinocilia of different hair cells are fused. (**I**) The apical pole of some hair cells are extruded into the statocyst cavity (black asterisks). Some hair cells have been totally ejected leaving holes on the sensory epithelium (white asterisks). Arrowheads signs to some hair cells that have lost the bundle of kinocilia. (**J**) A large section of sensory epithelia presents all its hair cells extruded above the epithelium (black asterisks). (**K**) Almost all the cell body of hair cells is ejected from a large region of the sensory epithelium (black asterisks). (**L**) Detail from (**K**) shows the cell body of the hair cells extruded. Scale bars: (**A**, **K**) = 30 μm. (**B**, **E**, **F**) = 10 μm. (**C, D**) = 5 μm. (**G–J, L**) = 20 μm.

Immediately after and 48 h after sound exposure (Fig. 2B–L) in comparison with the same tissues from control animals (Fig. 2A), damage was observed on the *macula statica princeps* sensory epithelium, by scanning electron microscopy (SEM) analysis. Immediately after sound exposure (Fig. 2B–H) there were spherical holes on the base of the hair cells and a rupture of the plasma membrane, probably due to the extrusion of the internal cellular material (Fig. 2D). Some hair cells had lost a number of kinocilia (Fig. 2E) or showed bent and flaccid kinocilia (Fig. 2B). The microvilli were flaccid and disorganized in all samples (Fig. 2B). In some cases the bundle of kinocilia of the hair cells are completely fused (Fig. 2C). The hair cells were partially (Fig. 2F–H) or totally ejected (Fig. 2F) from the sensory epithelium; in the latter case the holes left on the epithelium are visible (Fig. 2F).

In animals sacrificed 48 h after sound exposure (Fig. 2I–L), the sensory epithelium of the *macula statica princeps* presented the same lesions but theirs gravity and extension increased with time. Hair cells partially or totally ejected from the sensory epithelium are visible (Fig. 2H–I), leaving holes on the epithelium ejected (Fig. 2I). The apical ciliated apex and part of the cellular body were extruded above the sensory epithelium into the statocyst cavity (Fig. 2I–H). Some hair cells had totally, or in a considerable number, lost the kinocilia (Fig. 2I) and others exhibited bent kinocilia. In some regions almost the total cellular body is visible in the process of extrusion of the sensory epithelium (Fig. 2K,L).

**Figure 3:**
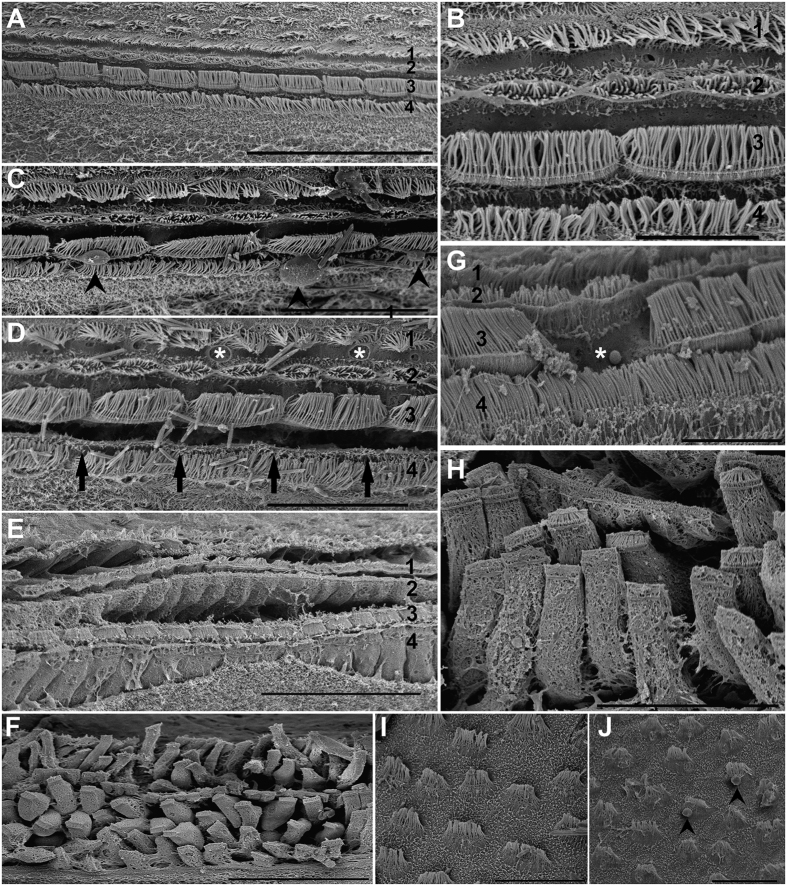
SEM.(A–H) *S. officinalis crista.* I, J: *S. officinalis macula neglecta superior*. A, B, I: control animals. (**C**–**E**), (**G**,**H**) sacrificed immediately after sound exposure. (**F**,**J**) sacrificed 48 h after sound exposure. (**A**) The four rows of hair cells of the *crista* of *S. officinalis* are visible. The hair cells from two of them are larger (3, 4) than those of the other two rows (1, 2). Surrounding the *crista*, other hair cells are found. (**B**) Detail of A showing the different structure of the four crista rows. (**C**) Row 2 shows cytoplasmic blebs (arrowheads) on the hair cells because of the inner cellular expulsion material. (**D**) The epithelium is fractured between rows 3 and 4 (arrows). The hair cells on the row 3 are partially extruded. Note that hair cells in row 1 have some holes in the basal part (asterisks). By contrast, kinocilia on hair cell show a healthy aspect amongst the four rows. (**E**) Hair cells, on the four rows show obvious signs of damage including bending kinocilia. The epithelium is fractured between row 2 and row 3. Note that hair cells in row 1, 2 and 4 are partially or totally extruded. (**F**) The more severe alterations in crista are shown. The epithelia is totally unstructured and the rows of hair cells are ejected into the cavity independently of the neighbouring cells, losing its original perfect alignment. Hair cells show loss or bent, flaccid or fused kinocilia. (**G**) A hair cell of the row 3 has been totally extruded into the statocyst cavity (asterisk). (**H**) Detail of the totally unstructured epithelium with totally extruded hair cells. (**I**) The hair cells on the *macula neglecta superior* are fewer and more separate that in the *macula statica princeps*. (**J**) Some hair cells of *the macula neglecta superior* show inner cell material extruding into the statocyst cavity by cytoplasmic blebs (arrowheads) and bent and flaccid kinocilia on the apical pole after sound exposure. Scale bars: (**A, E, H**) = 50 μm. (**B, G**) = 10 μm. (**C, D, I, J**) = 20 μm. (**F**) = 100 μm.

In *macula neglecta superior* and *macula neglecta inferior* (the two smaller units of the macula-statocyst system) epithelia we observed as a starting point of the damaging process 48 h after sound exposure in comparison with the same epithelia on the control animals (Fig. 3I). Some hair cells were seen extruding cytoplasmic material into the statocyst cavity (Fig. 3J) via cytoplasmic blebs and presented bent and flaccid kinocilia.

#### Crista epithelia

The normal organization of the crista epithelia from control animals is shown in Fig. 3A,B. In sound exposed animals, damage on crista epithelia was observed just immediately after sound exposure (Fig. 3C–E,G,H) and up to 48 h (Fig. 3F). Spherical holes (Fig. 3C) could be noticed at the base of the hair cells arranged in rows, as well as rupture of the plasma membrane (Fig. 3D,E) due to the extrusion of the inner cellular material by cytoplasmic blebs (Fig. 3C). As a consequence, apical poles of hair cells were partially or totally extruded (Fig. 3G) from the sensory epithelium. Some individuals showed bent and flaccid or lost kinocilia in their hair cell rows (Fig. 3E). The most damaged epithelia, presented hair cells completely extruded into the statocyst cavity independently of the neighbouring cells (Fig. 3F,H). The epithelia are completely unstructured and the rows of hair cells are ejected into the cavity losing its original perfect alignment (Fig. 3F,H).

### Image and data analysis

**Figure 4:**
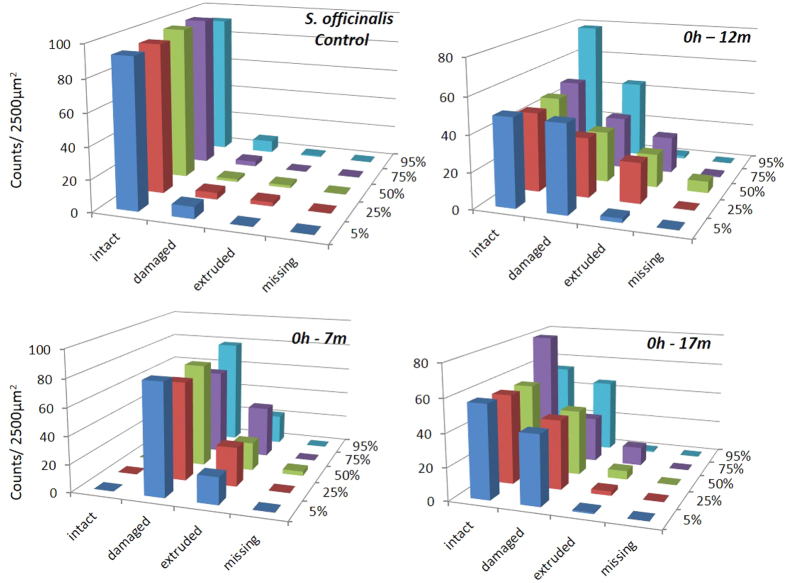
Mean intact hair cell, damaged hair cell, extruded hair cell and missing hair cell at 5, 25, 50, 75 and 95% of the total length of *macula statica princeps* of *S. officinalis* (0 h after sound exposure at 7 m, 12 m and 17 m of depth). Note the increase of damage, extruded and missing cells versus control animals with decreased distance to transducer.

**Figure 5:**
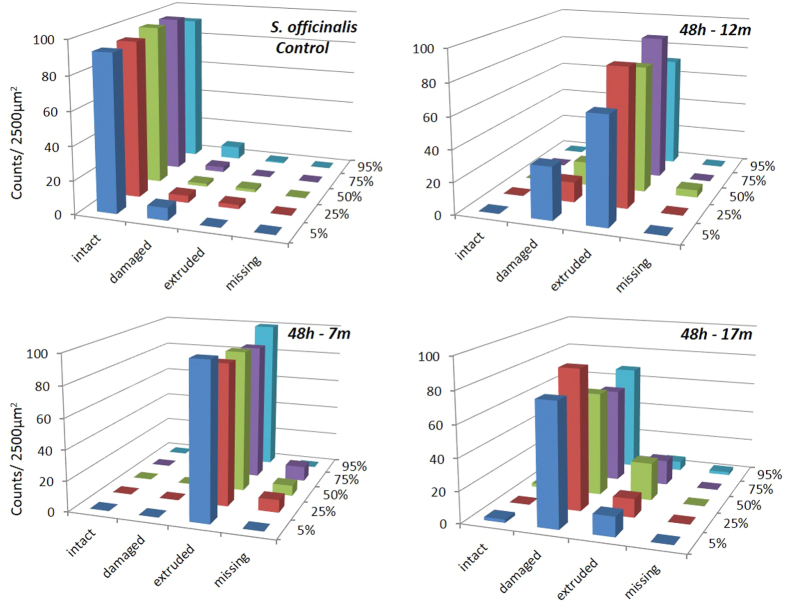
Mean intact hair cell, damaged hair cell, extruded hair cell and missing hair cell at 5, 25, 50, 75 and 95% of the total length of *macula statica princeps* of *S. officinalis* (48 h after sound exposure at 7 m, 12 m and 17 m of depth). Note the increase of damage, extruded and missing cells versus control animals with increase of time (by comparison with Fig. 4) and decreased distance to transducer.

The abnormal features we identified on the surface of sound-exposed *macula statica princeps* epithelium included damaged hair cell (with fused kinocilia and partially or entirely missing bundles), extruded (hair cell partially extruded from the epithelium) and missing hair cell (hole in the epithelium caused by the total extrusion of the hair cell). The number of damaged, extruded and missing hair cells was counted for each image. These numbers increased with time and decreased with the distance to the transducer (Figs 4 and 5).

**Figure 6:**
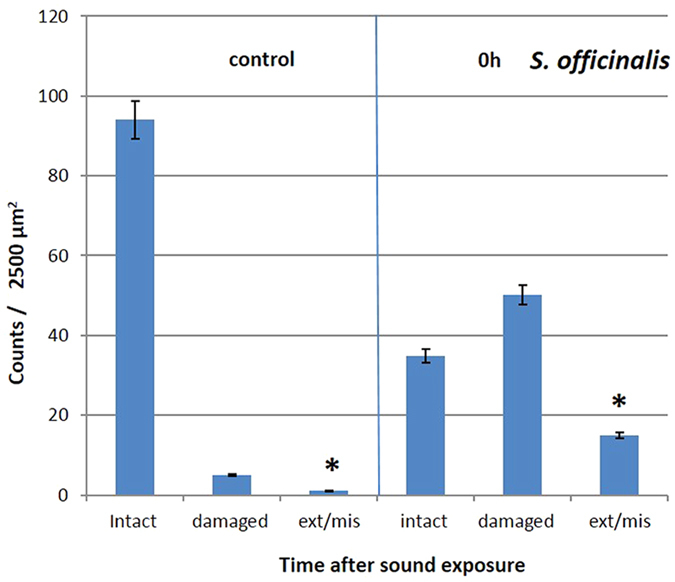
Mean ( ± SE) intact hair cell, damaged hair cell, extruded/missing hair cells on *macula statica princeps* epithelium of *S. officinalis*, 0 h after sound exposure versus control (*p = 0,002). Each bar is the average over the 5 zones with the line indicating the standard error. The percentage was computed by dividing with the total count for each individual sample.

The lesions were quantified as the percentage of extruded and missing hair cells because these are well-defined categories easier to compare. The presence of extruded cells determined the threshold of severe lesion after sound exposure. After statistical tests, the impairment quantification showed there were effects on *macula statica princeps* epithelium after exposure to the sound stimuli (p = 0,002) (Fig. 6).

**Figure 7:**
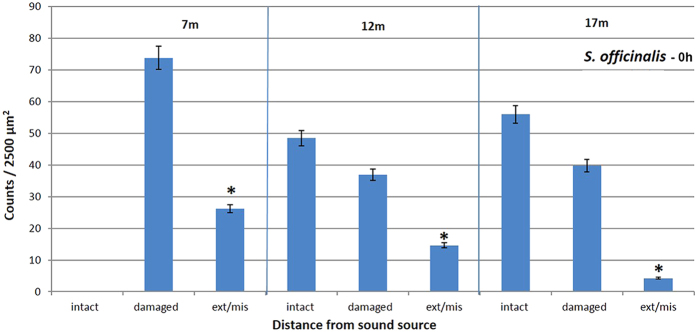
Mean (±SE) intact hair cell, damaged hair cell, extruded/missing hair cells on *macula statica princeps* epithelium of *S. officinalis*, 0 h after sound exposure, in function of the distance to the transducer. Permutation test 7 m–12 m (*p = 0.005); permutation test 7 m–17 m (*p = 0.005); permutation test 12 m–17 m (*p = 0.001). Each bar is the average over the 5 zones with the line indicating the standard error. The percentage was computed by dividing with the total count for each individual sample.

We determined that the distance to the transducer after exposure had effects on the level (severity) of the lesions in *S. officinalis macula statica princeps* epithelium (Fig. 7). The level of lesions was quantified as the number of extruded/missing hair cells at 0 h after sound exposure that decreased with distance. Kruskal-Wallis test showed that the three groups (placed at 7 m, 12 m and 17 m depth) do not share the same median (p = 0.007): permutation test between 7 m and 12 m (p = 0.005); permutation test between 7 m and 17 m (p = 0.005); permutation test between 12 m and 17 m (p = 0.001).

Although we appreciated an evident quantitative increase in the extension and severity of the lesions in the *macula statica princeps*, we did not statistically test the variables on the animals sacrificed 48 h after sound exposure because of the impossibility to control the environmental conditions effects on the animals while being held in cages at sea, in the water column.

### Particle motion measurements

**Figure 8:**
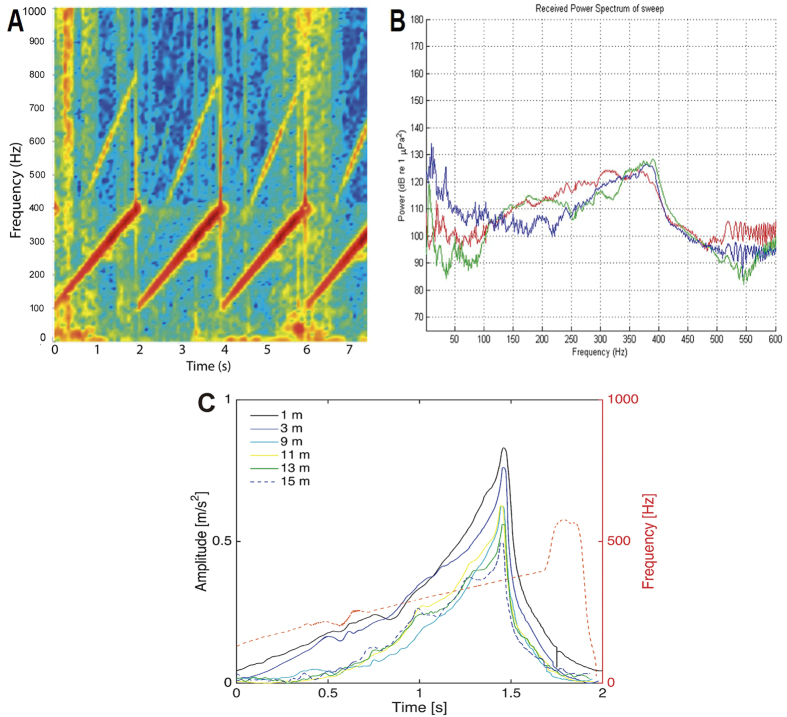
(**A**) Spectrogram of the sweeps (**B**) Power spectrum of the sweeps on all channels averaged over 8 seconds (4 sweeps) with a 1 Hz resolution. (**C**) The total amplitude and frequency for the transmitted pulse calculated by using the Hilbert transform. The amplitude is increasing with time and decreasing with distance. The frequency of the pulse is increasing linearly with time. At the end of the pulse there is a sudden increase of frequency above 400 Hz. The amplitude is however low.

On the experiment site, the particle motion sensor was kept suspended from the side of one of the platforms and placed at 7 m horizontal distance from the source. The depth measured from the sea surface of the particle sensor varied between 1 m and 15 m. The amplitude of the particle motion was obtained at 1, 3, 9, 11, 13 and 15 m depth. The results from 5 m depth were erroneous and are not dealt with here. The sound source generated linearly increasing frequency chirps, starting at 100 Hz and ending at 400 Hz. The output of the source was somewhat distorted resulting in overtones, which were clearly discernible at the end of the chirp, see Fig. 8C. However, the amplitude of the high frequency distortion was low and thus the impact on the hearing can be assumed to be negligible. The analysis showed that the time base of the source was highly accurate, thus, allowing extraction and analyses of single pulses. The total motion was calculated by summing the three orthogonal components of the acceleration. The Hilbert transform was used to determine the time dependence of both the frequency and the amplitude. These two processes were applied to the individual pulses and resulted in an ensemble of amplitudes and frequencies, which finally were stacked and median filtered (a filter length of 0.035 s was applied) to smooth out the fluctuations caused by rocking of the boat that was transferred to the sensor through the rope. The observed particle motion levels are shown in Fig. 8C. The maximal level was 0.7 ms^−2^ observed at 1 m depth (corresponding to a distance of 7.1 m between source and cage). The results are in line with what to be expected: the amplitudes were observed to decrease with increasing depth. The levels are relative high and the difference between 1 m and 15 m was about a factor of 2.

## Discussion

Sound effects on fish behaviour, hearing, and overall physiology are well documented^1^,^6^,^8^. Low intensity sounds, such as those produced by shipping are continuous, increasing and pervade a whole environment. For a fish, it is difficult to escape from a general increase in background noise. The studies that examined the effects of long-term noise exposure on fish^41^,^42^,^43^ showed that fish species that are sensitive to sound might show temporary hearing loss when exposed to increased background noise levels, whereas fishes with no sensitivity to sound do not necessarily show hearing loss. Numerous papers about the behavioural responses of fishes to marine vessel sound^44^,^45^,^46^,^47^,^48^,^49^ indicated that fishes typically exhibit some level of reaction to the sound of approaching marine vessels, the degree of reaction being dependent on a variety of factors including the activity of the fish at the time of exposure (e.g. reproduction, feeding, and migration), the characteristics of the vessel sound, and water depth. Other studies, which addressed the need of population assessment and a better management of fishing under the scope of the industry interests, also showed the effects of boat noise on fishes in the marine environment^49^,^50^,^51^. It was demonstrated that fishes actively avoid specific kinds of vessels by vertical and horizontal displacements. In addition to behavioural reaction, noise can elicit an endocrinological stress response in fish as well. Previous results^52^,^53^,^54^ reported changes in cortisol and other biochemical parameters in fishes after exposure to noise. The general consensus is therefore that ship noise that is characterized by amplitude and frequency fluctuations, constitutes a potential stressor to species possessing excellent hearing abilities and species with poor hearing abilities^55^. Some additional studies reported pathological effects of sound on sensory cells in the ears of some fish species^6^,^7^,^8^. This visual manifestation of damage could hide a much greater effect, a temporary inability to respond to the presence of predators or to locate preys and mates. However, no study has yet determined the correlation between damage of hair cells and permanent hearing loss in fishes.

The literature about sound effects on marine invertebrates^9^,^10^,^11^,^12^,^13^,^14^,^15^,^16^,^17^ and, in cephalopods in particular, is even scarcer, especially to what refers to pathological effects on sensory epithelia. Our previous laboratory experiments^18^,^19^,^20^ indicated acoustic trauma in sound-exposed cephalopods located in the statocysts, but the question remained on the influence in the results of the experimental conditions and no quantification of the lesions was performed.

This study included particle motion measurements, an unprecedented initiative aimed at determining its influence, versus or in combination with acoustic pressure, on the onset of the lesions. The measured amplitudes were observed to decrease with increasing depth but remained relatively high along the water column. In a controlled tank experiment made by Andre *et al*.^37^. *S. officinalis* was exposed to loud sound, utilizing laser Doppler vibrometer. The animals were anesthetized to keep it in in a fixed position while exposed to sound. The measured vibration of the neutrally buoyant animals was compared to a reference target. Both showed the same levels of acceleration in the sound field, strongly indicating that the animal’ body was vibrating in concert with the sound field. It could not, however, describe the complex interaction between sound field, wall vibrations and the presence of the cuttlefish. Yet, it could be established that the whole body was vibrating in the tank with a constant amplitude. Damages in the sensory epithelium were observed demonstrating that, in terms of vibration transmission, there was a direct correlation between the statocyst and the animal, causing the observed lesions. Nevertheless, it could not be ruled out that the configuration of the experiment (including a small tank) had no influence on the acoustic trauma. In the present *in-situ* offshore study the caged cuttlefish were exposed to levels of particle motion comparable to what was observed in the tank experiment. The wavelength of the sound was spanning from 1 m (400 Hz) to 3.5 m (100 Hz), thus, shorter than the typical dimension of the experimental site (depth of 25 m). It should be underlined that the uncertainties with the sound field that were prevailing in the tank were to a large extent removed. The observed levels of particle motion (see Fig. 8C) confirmed that the cuttlefish were located in a sound field that was homogenous over the animal’s body. This implies that the caged cuttlefish were moving as a solid-body in the sound field and that it was this motion that was transmitted to the statocyst and caused the damages. The nature of the quantified lesions, e.g. damaged, extruded and missing hair cells in the two experiments was similar, thus confirming that the animals in the tank experiment were vibrating as a solid-body. A first conclusion at hand is that the tank experiment offers an accurate and alternative methodology to study acoustic trauma on neutrally buoyant animals; free from the cumbersome logistics associated with *in-situ* offshore studies.

In the current offshore noise exposure comparative experiments on common cuttlefish (*Sepia officinalis*), SEM revealed injuries on the *macula* and *crista* epithelia of the statocysts. Exposure to the 100–400 Hz sinusoidal wave sweeps produced significant hair cell bundle extrusion and loss in the two studied sensory epithelia. The affected hair cell presented bent, flaccid or missed kinocilia. These results are the first to unequivocally demonstrate the sensitivity to noise of cephalopods in their natural habitat, affecting the exposed animals at physiological and pathological levels, and probably altering sound perception mechanisms and compromising their behaviour and capacity of survival in their natural habitat^56^. The damage, which we quantified as the percentage of extruded and missing hair cells, increased with time after sound exposure and decreased with the distance to the sound source. This indicates that threshold received levels can be established to help regulation addressing ocean noise issues on invertebrates.

The lesions were here quantified versus received noise levels and particle motion measurements. The analysis did not consider direct measured threshold levels at discrete frequencies. The choice of using the same experimental noise source as included in the laboratory CEE protocol^18^,^19^,^20^, i.e. a broadband sweep of frequencies covering the species sound sensitivity, was directed by the requirement to compare and validate our previous results before precisely determining which frequency and amplitude were responsible of the trauma. Acknowledging the validity of an experimental approach in laboratory conditions, this will constitute the next step of this research.

However, from Fig. 8B that represents the received power spectrum of the sweep used during the experiments, it was possible to extract the received levels at 1/3 octave frequency bands. Given the unreliable response of the transducer at very low frequencies, we determined that the animals were exposed at levels ranging from 139 to 142 dB re 1 μPa^2^ and from 139 to 141 dB re 1 μPa^2^, at 1/3 octave bands centred at 315 Hz and 400 Hz, respectively. Cuttlefish audiogram is not known but ABR experiments on *Loligo pealeii*^32^, another decapodiforme cephalopod, showed sensitivity at 400 Hz up to 140 dB re 1μPa SPL. The above received levels can therefore be considered a reasonable threshold estimation of noise levels that can trigger acoustic trauma in cephalopods.

## Methods

### Cephalopod specimens

Eighteen individuals from *S. officinalis* (mantle length 12–20 cm) were used immediately after being caught by experienced fishermen off the Catalan coast (NW Mediterranean Sea). They were maintained for a few hours at sea, in the same gear used for cephalopod collection, until performing the Controlled Exposure Experiments.

Several specimens (see below) were used as controls and were kept in the same conditions as the experimental animals until being exposed to noise.

### Ethics

No specific permissions were required for this location and research activity. The cuttlefish, a commercial species, were caught by local fishermen using traditional methods.

The experimental protocol strictly followed the necessary precautions to comply with the current ethical and welfare considerations when dealing with cephalopods in scientific experimentation^57^,^58^. This process was also carefully analysed and approved by the Ethical committee for scientific Research of the Technical University of Barcelona (UPC) and by the Ethical Committee of the EU FP7 project AQUO^59^.

### Sound Exposure Protocol

Sequential Controlled Exposure Experiments (CEE) were conducted on adult individuals. A set of 9 individuals was used as a control: 3 before the exposure, 3 at 0 h and 3 at 48 h after sound exposure. The same sequential CEEs were conducted as with other cephalopods spp. studied in previous experiments^20^,^21^. The difference here is that, since the results from the analysis with *S. officinalis* showed lesions immediately after noise exposure, and incremental effects up to 96 hours (longest period of observation), we concentrated the study on animals sacrificed immediately after and 48 hours after exposure, thus reducing the number of specimens used in the experiments.

**Figure 9:**
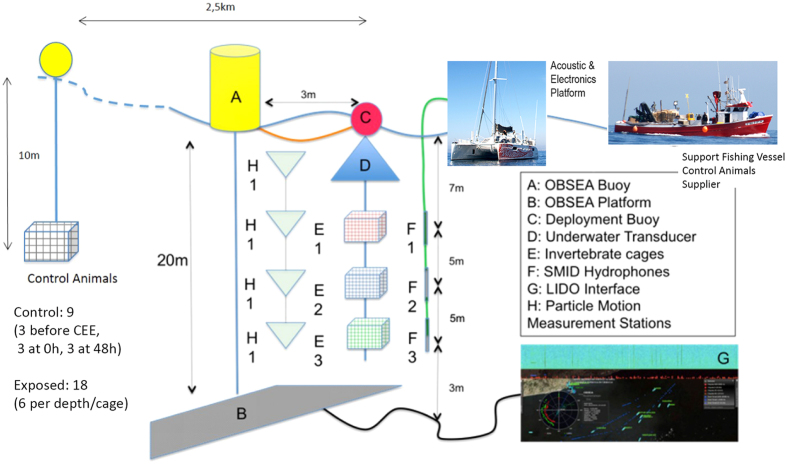
Scheme of the general protocol of the exposure to sound and posterior analyses.

**Figure 10:**
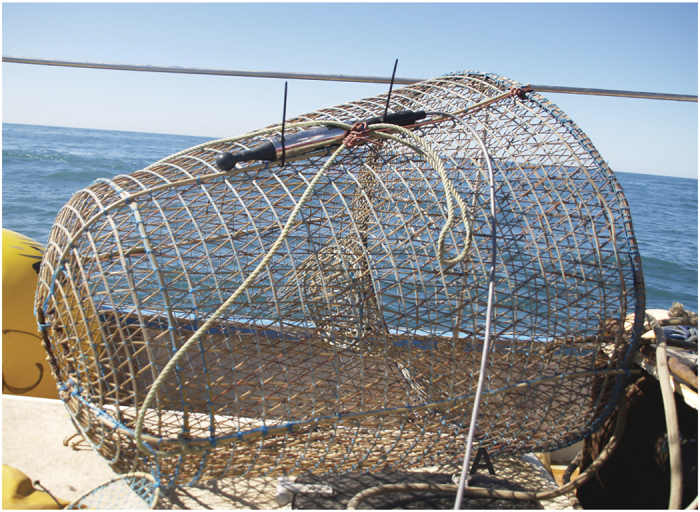
Position of the SMID hydrophone on the cage.

The exposure consisted of a 100–400 Hz sinusoidal wave sweeps with 100% duty cycle and a 1-second sweep period for two hours (Fig. 8A). The sweep was produced and amplified through a Lubell LL-1424HP Transducer, while the received level was measured at each depth by a calibrated SMID hydrophone (Figs 9 and 10). The cages with the animals were placed at 7 m, 12 m and 17 m depth (Fig. 9) close to the OBSEA platform in front of our laboratory in Vilanova i la Geltrú (Barcelona, Spain). Some of the animals were used as controls and were kept in the same conditions as the experimental animals until the latter were exposed to noise, in an independent trap 2, 5 km away from the experiment site.

### Particle motion measurements

Particle motion measurements were conducted at the same locations and depths (7 m, 12 m, 17 m) where the individuals were exposed to sound. The total amplitude and frequency were calculated using Hilbert transform. Particle motion levels were measured using the principle of a vibrating neutral buoyant sphere sensitive to acceleration. The design and the principles of the sensor was described in Sigray 2011^60^, but the diameter of the sphere used in this study was 0.06 m. The accelerometer mounted inside the sphere was triaxial and manufactured by PCB Piezoelectronics Inc., (model 356B18). Prior to the measurement the sensor was calibrated to assure that the results were both accurate and reliable.

### Removal of statocysts

After the exposure, the individuals that were not immediately sacrificed were placed in traps at sea, at the same location where the experiments were performed. The control animals were moved to adjacent traps. Following exposure, the samples were obtained from the individuals (exposed and controls) at 0 h and 48 h after sound exposure.

Contrary to what was stated in previous publications that presented results in laboratory conditions^18^,^19^,^20^, the current experiment was designed to quantify lesions versus received levels and thus estimate threshold levels that could trigger acoustic trauma in *S. officinalis* in offshore conditions.

In all experiments, isolated head preparations were obtained by decapitation. The statocysts with their surrounding cartilage were extracted and fixed for observation and analysis. For fixation, the statocyst cavity was opened and special care was taken to prevent mechanical damage to the inner tissues. The analysis was performed on tissues obtained from left and right statocysts.

### Imaging Techniques

The same imaging techniques were used as in previous works^20^,^21^. Individuals were processed according to routine SEM procedures.

### Scanning electron microscopy

Fifty four statocysts from 27 *S. officinalis* were used for this study. Fixation was performed in glutaraldehyde 2, 5% for 24–48 h at 4 °C. Statocysts were dehydrated in graded alcohol solutions and critical-point dried with liquid carbon dioxide in a Leica EmCPD030 unit (Leica Mycrosystems, Austria). The dried statocysts were cut, open and flattened out to expose the statocyst structures and then mounted on specimen stubs with double-sided tape. The mounted tissues were gold-palladium coated with a Polaron SC500 sputter coated unit (Quorum Technologies, Ltd.) and viewed with a variable pressure Hitachi S3500N scanning electron microscope (Hitachi High-Technologies Co., Ltd, Japan) at an accelerating voltage of 5 kV in the Institute of Marine Sciences of the Spanish Research Council (CSIC) facilities.

### Quantification and Data analysis

**Figure 11:**
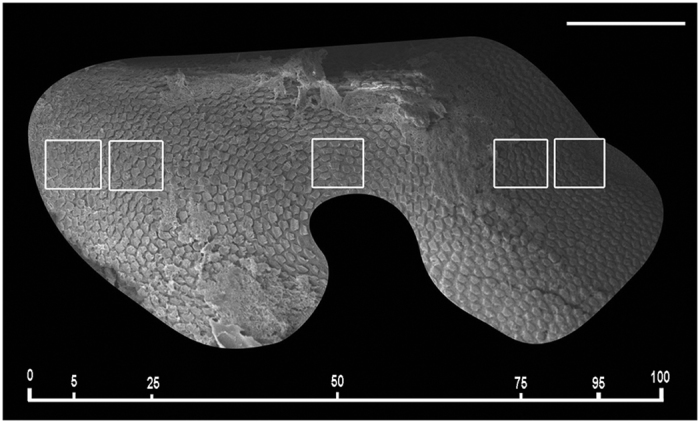
SEM. Sepia officinalis macula statica princeps. Hair cell bundles count locations on the *S. officinalis macula statica princeps.* Hair cells counts were sampled at five predetermined locations: 5, 25, 50, 75 and 95% of the total macular length. A 2500 μm^2^ box was placed at each sampling area and hair cells were counted within each box. Scale bar = 100 μm.

We considered for the quantification the region comprising the whole sensory area of the *macula statica princeps*. This structure was chosen because it is the biggest subunit of the *macula*-statolith system and due its anterior location and relative flat structure is the best to visualize the sensory epithelium. The length of the area comprising hair cells was determined for each sample, and 2500 μm2 (50 × 50 μm) sampling squares were placed along the centre length of the area at 5, 25, 50, 75 and 95% of the length axe of the macula statica princeps (Fig. 11).

To observe the presence of possible abnormal features on the surface of sound-exposed epithelia, as well as differences in hair cells appearance, hair cell damage was analysed by classifying the hair cells as intact (hair cell undamaged), damaged (bundle of kinocilia partially or entirely missing, bent or fused), extruded (hair cell partially extruded of the epithelium) or missing (hole in the epithelium caused by the total extrusion of the hair cell).

Damage due to sound exposure was tested using permutation tests. Lesions were quantified as the percentage of extruded and missing hair cell. Data was summed over all regions. Permutation tests were repeated multiple times with N = 1000 (test groups: control animals vs exposed animals (0 h all distances). The influence of the distance to the transducer was tested using Kruskal-Wallis analysis of variance test and permutation tests were repeated multiple times with N = 1000 (test groups: exposed animals 0 h separated per distance).

## Additional Information

**How to cite this article**: Solé, M. *et al*. Offshore exposure experiments on cuttlefish indicate received sound pressure and particle motion levels associated with acoustic trauma. *Sci. Rep.* **7**, 45899; doi: 10.1038/srep45899 (2017).

**Publisher's note:** Springer Nature remains neutral with regard to jurisdictional claims in published maps and institutional affiliations.

## Figures and Tables

**Figure 1:**
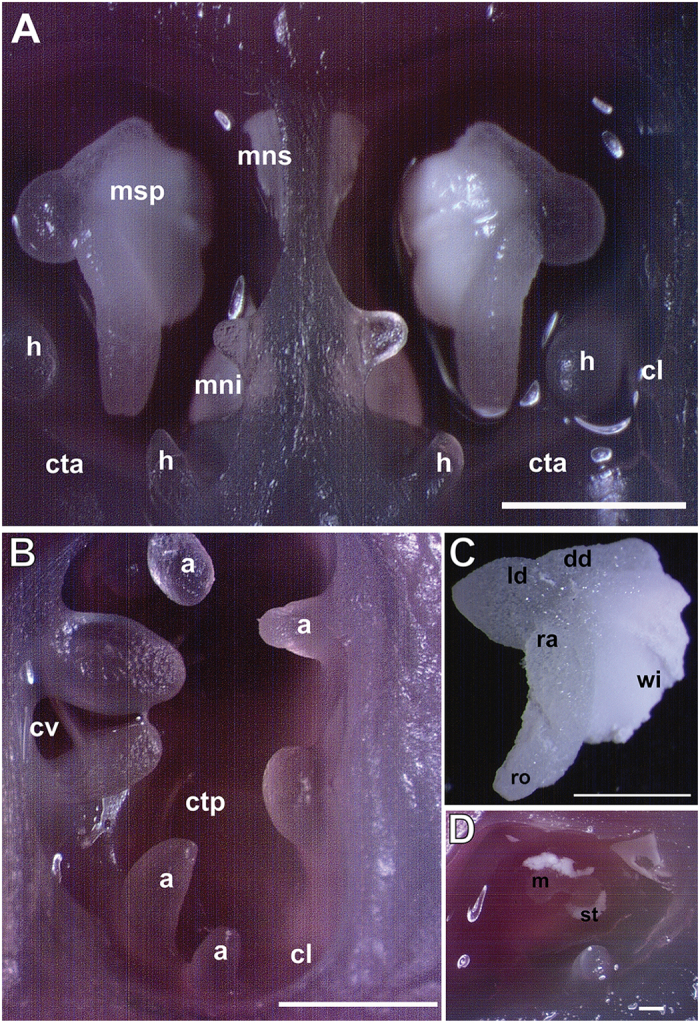
LM. Sepia officinalis statocyst structure. The statocyst cavities have been opened transversally. Photomicrograps. (**A**) Anterior view. Each cavity shows the three macula-statolith systems (msp, mns, mni) and two of the crista-cupula systems (cta, cl). (**B**) Posterior view of one of the cavities. The two posterior crista segments (ctv, cv) are showed. **(C**) *S. officinalis* statolith showing its parts. (**D**) The statolith has been removed and the *macula statica princeps* (m) is visible into the cavity. Some statolith traces are visible (a: anticrista lobe, cl: *crista longitudinalis,* cta: *crista transversalis anterior*, ctp: *crista transversalis posterior*, cv*: crista verticalis*, dd: dorsal dome, h: hamuli lobe, ld: lateral dome, mni*: macula neglecta inferior*, mns: *macula neglecta superior*, msp*: macula statica princeps,* ra: rostral angle, ro: rostrum, st: statolith, wi: wing). Scale bars: A, B = 2 mm. C = 1 mm. D = 200 μm.
